# The American Dental Society of Europe

**Published:** 1883-09

**Authors:** 


### THE AMERICAN DENTAL SOCIETY OF EUROPE.

Just in time for insertion in the last form, we have received the following letter, which explains itself. —EDITOR.

I have just returned from the meeting of the American Dental Society of Europe, at Cologne. The sessions lasted for three days, the 7th, 8th, and 9th of Aug., the same days on which the American

Dental Association met at Niagara. The meeting was one of unusual interest, and the attendance was the largest ever known, nearly every country in Europe being represented. Among the guests, who are always heartily welcomed, was Dr. Joseph Walker, President of the Odontological Society of Great Britain. * * * * *

Dr. Elliott's very valuable experiments on amalgams were especially interesting.

Dr. Jenkins, the champion of intelligent bridge work in Europe, was also there, and treated the Society to some very useful hints.

Dr. Miller was, of course, present, with a few of his preparations. He thinks he has, by hard work and purely scientific experiments, furnished a definite solution of the following points in the Etiology of Dental Caries:

1. The first step is an almost *complete* decalcification of the tissue by acids, resulting, in the case of enamel, in its entire destruction.

2. Following the decalcification, (often far behind it) is the destruction of the *decalcified* tissue by micro-organisms.

3. The fact that an artificial caries identical with natural caries may be easily produced, renders untenable the idea that inflammation is necessary to caries.

According to Dr. Miller, there remains to be solved the questions: *What is the acid or acids of caries? How is it produced? Do fungi produce or help to produce it? If so, what fungi, and how is their development in the mouth to be hindered?*

I do not propose, however, in this letter to give even a synopsis of the work done at the meeting. I hope within the next few days to send you a full report of the proceedings.

The latter part of the afternoon, and the evening of the second day, were devoted to an excursion, *en voiture,* along the banks of the Rhine, to a neighboring village, where a grand banquet had been prepared. You will recall a similar occasion two years ago.

The American dentists in Europe are hard workers, but they haven't forgotten how to have a good time, and when they set aside one evening in the year to the latter purpose it is apt to be a success. At least it was so on this occasion. The festivities were accompanied by music, illuminations, fireworks, etc., etc., and enough funny speeches were made to last for a year, and make us all long for the next annual feast, which is to be held among the mountains of Switzerland. The ceremonies were conducted by Dr. Patton, of Cologne, in a manner worthy of the reputation which he enjoys among the American dentists on this side of the great waters.

The following officers were elected for the year '83 and '84:

President—Dr. W. D. Miller, Berlin.

Vice President—Dr. H. C. Edwards, Madrid.

Secretary—Dr. F. Forster, Berlin.

Treasurer—Dr. Wm. Patton, Cologne.

Microscopist—Dr. W. D. Miller, Berlin.

